# Bladder metastasis from primary breast cancer: a case report

**DOI:** 10.1186/s40792-018-0484-6

**Published:** 2018-07-09

**Authors:** Kimiyasu Yoneyama, Motohito Nakagawa, Asuka Hara

**Affiliations:** Department of Breast Surgery, Hiratsuka City Hospital, 1-19-1 Minamihara, Hiratsuka-shi, Kanagawa 254-0065 Japan

**Keywords:** Breast cancer, Metastasis, Urinary bladder

## Abstract

**Background:**

Breast cancer frequently metastasizes to the bone, lung, and liver. However, metastasis to the bladder is uncommon. Bladder metastasis due to direct infiltration from peripheral organs, such as the colon and rectum, prostate, and cervix, occurs more frequently than metastasis from distant organs, such as the breast.

**Case presentation:**

We report a case of bladder metastasis identified during treatment for recurrent breast cancer. Fifteen years after her initial surgery, a known breast cancer patient complained of a left lower abdominal pain, anuria, and body swelling. Computed tomography imaging revealed an irregular thickening of the left bladder wall, left hydronephrosis, and hydroureter. A bladder metastasis from breast cancer was diagnosed based on a histological examination of a cystoscopic biopsy specimen. She is currently receiving chemotherapy with eribulin mesylate.

**Conclusions:**

Routine screening of the lower urinary tract is not necessary for all patients, but women with a history of breast cancer presenting with urinary symptoms should undergo a thorough examination of the urinary tract.

## Background

Secondary bladder tumors are rare and comprise only 2% of all bladder tumors, most of which are found at autopsy [1, 2]. Primary tumors that cause secondary bladder carcinoma include stomach cancer, malignant melanoma, breast cancer, and lung cancer. Breast cancer generally metastasizes to the lymph nodes, lungs, liver, bones, and brain. Metastatic lesions in the adrenal gland, spleen, thyroid, heart, and bladder are uncommon. In general, bladder metastasis from distant organs is also rare, but direct infiltration from cancer in surrounding organs, such as colorectal, prostate, and cervical cancer, occurs more frequently. We report a case of bladder metastasis identified during treatment for recurrent breast cancer.

## Case presentation

**Fig. 1:**
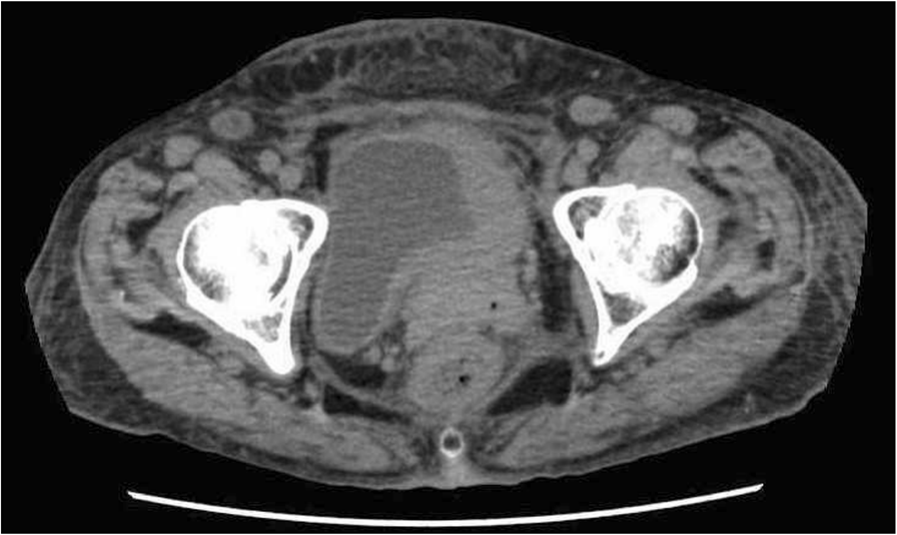
Computed tomography. Thickening of the left bladder wall

**Fig. 2:**
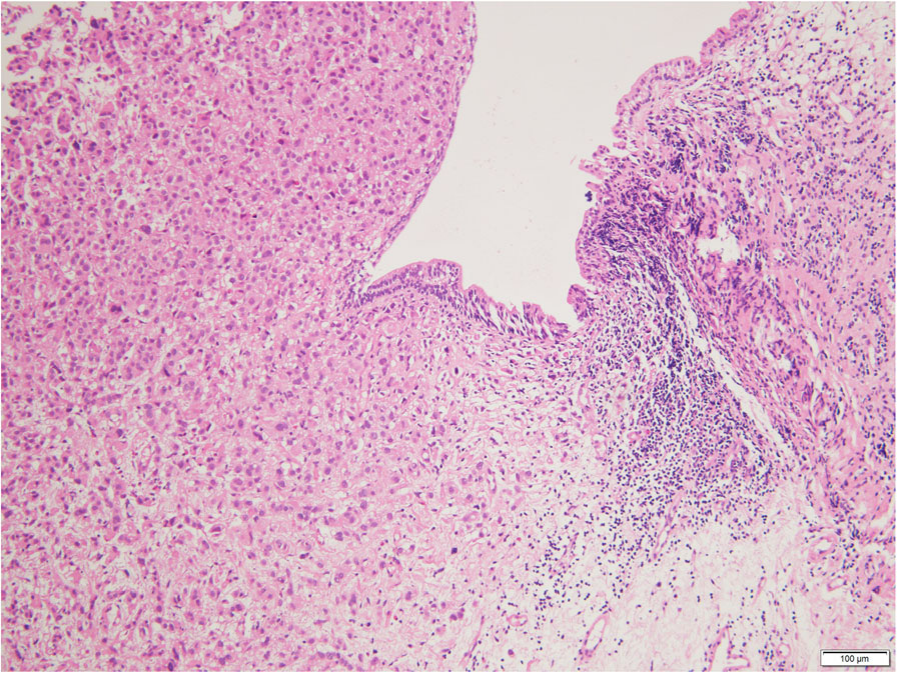
Histopathology. Eosin-stained cells with a large nucleus are seen proliferating in the lamina propria mucosa

**Fig. 3:**
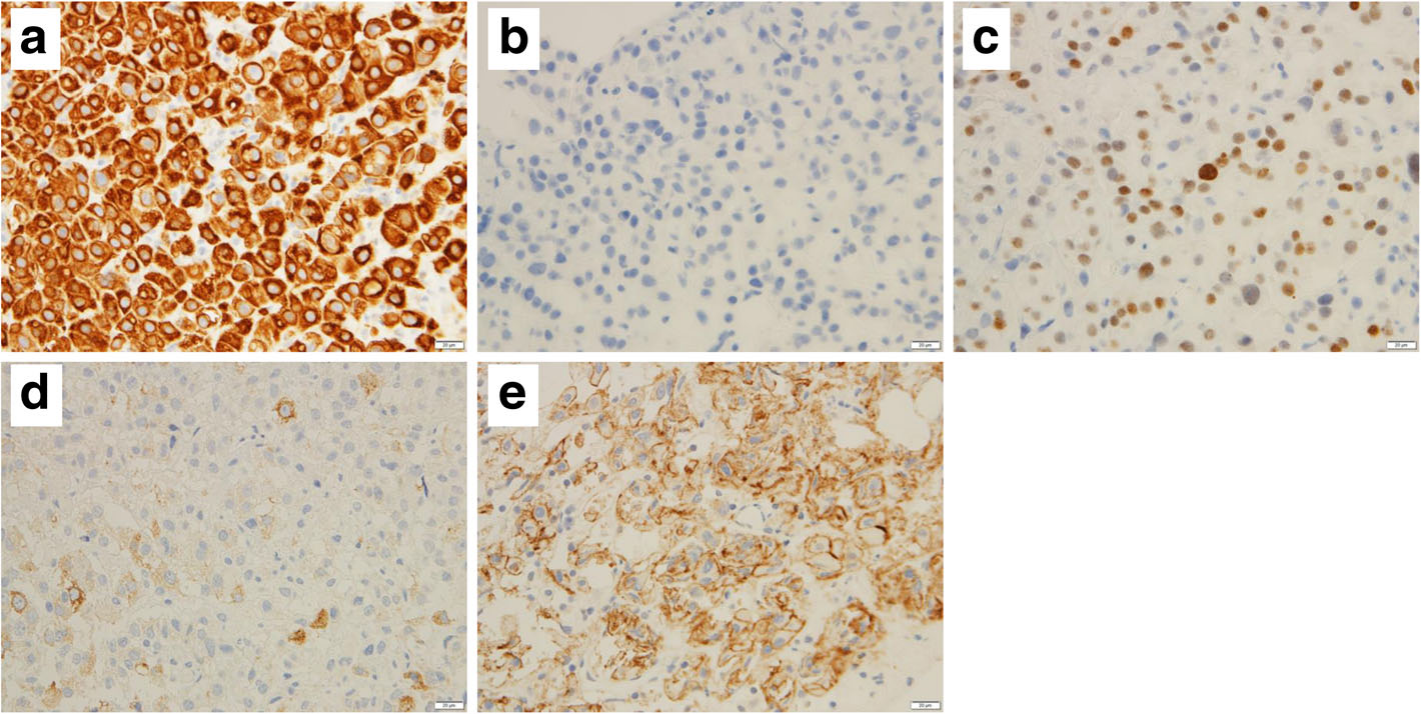
Immunohistochemical findings. Immunohistochemically, the tumor cells were positive for CK-7 (**a**) but negative for CK-20 (**b**). ER (**c**), GCDFP-15 (**d**), and HER2 (**e**) were positive. All scale bars, 20 μm

A 68-year-old woman underwent a left mastectomy and axillary lymph node dissection for left breast cancer (T4bN2M0). A pathological examination revealed an estrogen receptor-positive (ER-positive), progesterone receptor-positive (PgR-positive), and human epidermal growth factor receptor 2-positive (HER2-positive) invasive ductal carcinoma. For postoperative therapy, 6 cycles of 5-fluorouracil+epirubicin+cyclophosphamide and oral tamoxifen were given. A right renal cell carcinoma was incidentally noted on computed tomography imaging performed at follow-up 2 years later, and a right nephrectomy was performed. A further 4 years later, a bone biopsy was performed for a suspected bone metastasis found at the distal end of the left femur. This lesion was diagnosed as a metastasis from the primary breast cancer. Since the bone metastasis was localized within a single site, radiation therapy to this site and high-dose toremifene therapy were administered. Fifteen years after the initial surgery, she developed a left lower abdominal pain, anuria, and body swelling. Computed tomography imaging revealed an irregular thickening of the left bladder wall, left hydronephrosis, and hydroureter (Fig. 1). As the ureteral orifice was occluded, an urgent left nephrostomy was immediately performed. A cystoscopy revealed a broad-based tumor extending from the left wall to the triangle of the bladder. The ureteral orifice could not be identified. The tumor was biopsied, and a histopathological examination revealed a proliferation of cells with eosinophilic cytoplasm and a rounded dentate macronucleus in the mucosal lamina propria (Fig. 2). The immunostaining results revealed CD7 positivity, CD20 negativity, ER positivity, and HER2 positivity, confirming a diagnosis of bladder metastasis from breast cancer (Fig. 3). High-dose toremifene was considered ineffective, and chemotherapy with eribulin mesylate was started.

## Discussion

The first report of bladder metastasis from breast cancer was by Haid et al. [3] in 1980, but the first autopsy report was published in 1956 [4]. To date, there have been about 50 reported cases of metastatic breast cancer to the bladder [5]. Bladder metastasis and retroperitoneal metastasis are considered to occur more frequently with invasive lobular breast carcinoma. The metastatic pathway could be hematogenous, lymphogenous, or direct retroperitoneal invasion.

The most common symptoms of bladder carcinoma are frequent urination and gross hematuria. Other clinical features include difficulty in urination, ureteral obstruction or urinary incontinence, a pelvic mass, bilateral hydronephrosis, and ultimately renal failure. Bladder carcinoma can also present as an incidental finding in imaging studies. Some cases are found simultaneously with the primary tumor or after a long course of more than 30 years [6]. On the other hand, some cases of breast cancer are diagnosed after discovering a bladder metastasis. In most cases, the cancer has already become widespread at the time of diagnosis, and it is rare for only bladder metastasis to be detected, as in our case [7]. Screening with magnetic resonance imaging and positron emission tomography–computed tomography is useful for the diagnosis of metastasis to other sites. In the case of renal dysfunction with suspected obstructive nephropathy, an examination of the upper urinary tract is also necessary. To confirm the diagnosis, observation of the bladder mucosa by cystoscopy and histological examination of a specimen obtained by biopsy or transurethral resection are necessary. Cystoscopic findings include obvious tumors, nonspecific inflammation, and a thickened bladder wall covered with normal mucosa. The presence of changes in the bladder mucosa is useful in distinguishing a primary bladder tumor from a metastatic bladder tumor. A submucosal tumor is suggestive of a secondary bladder tumor, but ulcerative lesions can be seen in some cases. In the present case, a diagnosis was difficult based on morphology alone, and an immunohistochemical analysis was necessary. Common screening markers for suspected breast tumors include the expression of cytokeratin, CK-7, CK-18, CK-19, CK-20, GCDFP-15, and ER/PgR. The treatment of metastatic breast cancer involves chemotherapy and hormonal therapy. Local resection is often performed for diagnostic purposes and to improve local symptoms. In our case, the immunohistochemical characteristics of the metastatic lesion were similar to those of the primary tumor. This was an important factor leading to a definitive diagnosis, in addition to the pathological results obtained using hematoxylin and eosin staining. However, not uncommonly, the ER/PgR status of the metastatic lesion can differ from that of the primary tumor, with one study reporting an inconsistency rate as high as 24% [8]. HER2-positive breast cancer tends to be more aggressive than other breast cancers and less responsive to hormonal therapy. If the patient has hydronephrosis because of a urinary obstruction, constructing a nephrostomy before the start of chemotherapy should help to improve renal function. The prognosis of secondary bladder carcinoma is very poor, and most patients die within 1 year. However, some cases with a survival of 5 years or more after diagnosis have been reported [9]. Bladder metastasis from breast cancer is often advanced at the time of diagnosis. It is recommended that accurate diagnosis be pursued and systemic therapy be started promptly. In our case, we confirmed the presence of a bladder metastasis from a primary breast carcinoma by confirming the presence of CD7 positivity, CD20 negativity, ER positivity, GCDFP-15 positivity, and HER2 positivity, in addition to the result of a pathological examination.

## Conclusions

Bladder metastasis from breast cancer is uncommon. Routine screening of the lower urinary tract is not necessary for all patients. However, for women with a history of breast cancer presenting with urinary symptoms, urinary tract examination to exclude bladder lesions should be performed. Appropriate treatment should be started as soon as a diagnosis is confirmed.

## Abbreviations

CD: Cluster of differentiation
CK: Cytokeratin
ER: Estrogen receptor
HER2: Human epidermal growth factor receptor 2
PgR: Progesterone receptor
